# Supplementary figures and images for: Effects of post oak (Quercus stellata) and smooth brome (Bromus inermis) competition on water uptake and root partitioning of eastern redcedar (Juniperus virginiana)
Source: PLoS One. 2023 Feb 1;18(2):e0280100. doi: 10.1371/journal.pone.0280100 (PMC9891534; doi:10.1371/journal.pone.0280100)

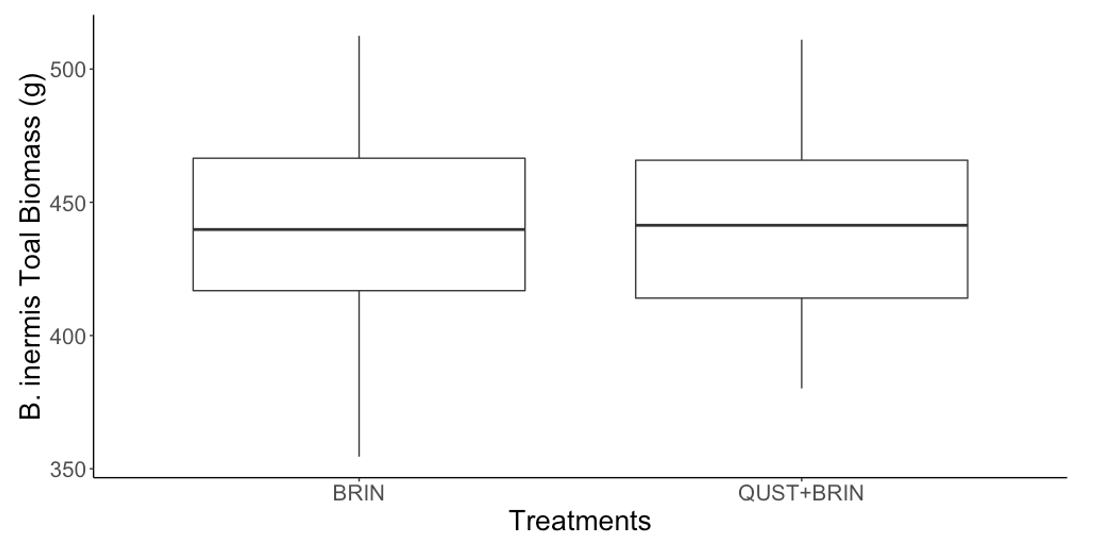

Supplement: S1 Fig — (TIF) [file pone.0280100.s001.tif]
