# Supplementary material for: Effects of post oak (Quercus stellata) and smooth brome (Bromus inermis) competition on water uptake and root partitioning of eastern redcedar (Juniperus virginiana)
Source: PLoS One. 2023 Feb 1;18(2):e0280100. doi: 10.1371/journal.pone.0280100 (PMC9891534; doi:10.1371/journal.pone.0280100)
Supplement: S1 Table — Cubic splines had higher coefficients of determination than the linear models. All regression lines were significant (p < 0.001). (PDF) [file pone.0280100.s002.pdf]

J. virginiana treatments	**Sep. 2018 Linear regression**	**Aug. 2019 Linear regression**	**Sep. 2018 Cubic spline**	**Aug. 2019 Cubic spline**
JUVI	y = 4.07-0.09x<br><br>$R^2 = 0.54$	y = 3.66-0.08x<br><br>$R^2 = 0.59$	$y = -8.67+1.83x-0.08x^2+1.04E-3x^3$<br>$R^2 = 0.87$	$y = 7.78+1.66x-0.08x^2+9.72E-4x^3$<br>$R^2 = 0.90$
QUST	y = 2.98-0.05x<br><br>$R^2 = 0.46$	y = 1.27-0.03x<br><br>$R^2 = 0.23$	$y = 1.76+0.18x-0.01x^2+1.79E-4x^3$<br>$R^2 = 0.50$	$y = 0.97+0.04x-3.73E-3x^2+5.81E-5x^3$<br>$R^2 = 0.25$
BRIN	y = 1.25-0.03x<br><br>$R^2 = 0.30$	y = 2.54-0.05x<br><br>$R^2 = 0.56$	$y = 1.91-0.1x+1.87E-3x^2-1.05E-5x^3$<br>$R^2 = 0.37$	$y =3.58-0.16x+2.61E-3x^2-1E-5x^3$<br>$R^2 = 0.63$
QUST+BRIN	y = 1.61-0.04x<br><br>$R^2 = 0.58$	y = 4.69-0.11x<br><br>$R^2 = 0.72$	$y = -2.36+0.56x-0.03x^2+3.28E-4x^3$<br>$R^2 = 0.79$	$y = -8.19+1.89x-0.09x^2+1.15E-3x^3$<br>$R^2 = 0.99$
