# Supplementary material for: Effects of post oak (Quercus stellata) and smooth brome (Bromus inermis) competition on water uptake and root partitioning of eastern redcedar (Juniperus virginiana)
Source: PLoS One. 2023 Feb 1;18(2):e0280100. doi: 10.1371/journal.pone.0280100 (PMC9891534; doi:10.1371/journal.pone.0280100)
Supplement: Attachment — Submitted filename: Response to Reviewers.docx [file pone.0280100.s003.docx]

PONE-D-22-20878

Effects of post oak *Quercus stellata* and smooth brome *Bromus inermis* competition on water uptake and root partitioning of eastern redcedar *Juniperus virginiana*

**Responses to the Reviewers:**

We thank the Editor and the Reviewers for their comments and suggestions to improve our manuscript.

We rearranged, modified, and shortened the different parts of the manuscript, including the Abstract, Introduction, and Discussion. We only included information pertinent to the interpretation of the results of our experiment.

We agree with the Reviewers that our design does not allow us to say from which depth water was extracted in the soil profile and by whom. Therefore, we modified our language in the Results and Discussion to predict and infer shift in water uptake at different depths, between treatments, and over two years.

We replaced ERC, post oak, and smooth brome with J. virginiana, Q. stellata, and B. inermis, respectively. In addition we replaced the ERC treatment with JUVI, ERC + post oak with QUST, ERC + smooth brome with BRIN, and ERC + post oak + smooth brome with QUST+BRIN.

Finally, we ensured that our manuscript meets PLOS ONE's style requirement.

**Reviewer #1: The authors conducted a greenhouse experiment to determine the rooting depth and water uptake across soil layers for ERC both growing alone and in competition with smooth brome and/or post oak. Measurements include root length, water status including midday leaf water potential and relative water content. Water sources and plant competition are very important topics in forest health and mortality under global climate change. However, some issues should be solved in the present version. Because there are few functional traits measured in this study, sufficient context drawn from other publications should be used to strengthen your work. The organization of the manuscript needs to be improved, the Introduction, Result, and Discussion should be shortened and condensed, the Methods should be strongly improved.

The Introduction could be shortened and integrated into four paragraphs, introduction to (1) competition, (2) the two-layer hypothesis, (3) ERC, smooth brome, and post oak, (4) purpose and hypotheses. Moreover, the introduction requires more cohesion and coherence between paragraphs even sentences, and the knowledge gaps should be emphasized.
The Methods should be strongly improved. Some details should be state more clearly. Showing photos related to the experiment can help readers understand your experiment design better.
The Result should be shortened, focus on the brief and scientific description of your results rather than discussing how your results came from. The results could be described one by one according to the figures and tables. Or, you can rearrange the Result according to the logical order of the manuscript.
The Discussion could be integrated into several parts, a subtitle should be given to each part to make the discussion clear. Your predictions should be mentioned and discussed here. In addition, some introductory sentences can be integrated with the Introduction.**

**Response**: We thank Reviewer#1 for helping us to clarify this manuscript. We have altered the manuscript as requested.

Details:

**Line 33: Change "very young plants" to "seedlings and saplings".**

**Response**: Altered as requested (L 87).

**Line 35–40: Although these are well known, new references need to be added herein. Please see a relevant paper of Liu et al. 2021, Frontiers in Plant Science, doi: 10.3389/fpls.2021.760510.**

**Response**: Thank you for the suggestion. Citation was added in L 71.

**Line 62–78: What is the root form of ERC, smooth brome, and post oak? In the wild, how long can the root of the three species reach? More root-related functional traits and relevant studies should be introduced here.**

**Response**: ERC has an extensive root system, which is deep and may penetrate 7 m into the soil. Post oak have taproots and can distribute their roots throughout the upper several meters of soil. Smooth brome has rhizomes for roots and can reach up to1.4 m in depth.

We have added this information as requested in L 107, 113, 114, 399, and 411.

**Line 112–113: What is the field capacity or saturated moisture content of the PRO-MIX? What are the similarities and differences between PRO-MIX and soil under ERC forest?**

**Response**: The PRO-MIX® has a medium-high moisture retention and a moisture content of 30 - 50 % by weight (L 162) .

Field capacity and saturated moisture content for soil under ERC forest is variable and depends on many biotic and abiotic factors. In addition, ERC can grow in many soils type, texture, and moisture content (Meneguzzo and Liknes, 2015). Therefore, to focus on studying the effects of competition on *J. virginiana* water uptake and root partitioning*,* we used potting mix.

**Line 121–129: When did the experiment start and finish? Why did the amount of water be controlled? Was it to simulate the local precipitation? In Fig. 1, the volumetric soil water content is lower than 4%, in my point of view, the soil water content in the experiment was so low that it was just like drought treatment. Were the three species faced with drought stress?**

**Response**: We ran the experiment in a greenhouse for two years from September 2017 until August 2019.We controlled the amount of water to avoid overwatering or underwatering the plants. The amount of water to add was determined using preliminary experiments. In addition, our water potential data shows that the plants were not water stressed (Fig. 5). We included this information in Discussion (L 465-466).

**Line 131: Too few functional traits were measured in this study. Did you measure the distribution pattern of roots, such as ratio of fine root to thick root, specific fine root area? If not, sufficient context drawn from other publications should be used to strengthen your work.**

**Response**: We did not measure the distribution pattern of roots; we only measured root length and weight. However, we included the below reference in the discussion: Ward, D. (2021). Shade affects fine-root morphology in range-encroaching eastern redcedars (*Juniperus virginiana*) more than competition, soil fertility and pH. *Pedobiologia, 84*, e150708.

**Line 132: Change "trunk diameter (at root collar)" to "basic diameter". How do you show your basic diameter? It is better to draw a figure to show the height and basic diameter in different periods**.

**Response**: We deleted this part from the Methods section because it was not relevant to the interpretation of the results of this manuscript.

**Line 161: Before ANOVA, was normality and homogeneity of variance tested? What statistical methods did you use to test normality and homogeneity? Please state clearly at the beginning of the Statistical analysis. Besides, the critical value (α) of the test should be mentioned.**

**Response**: We added the required information in L 219-221.

**Line 326–334: This part looks more like an introduction, maybe it is better to be integrated with the Introduction.**

**Response**: We shortened this part and moved it to the Introduction (L 53-57).

**Line 388: Here the authors made a comparison between the two years. Soil moisture is not only related to plants, but also to the weather. Was the weather of 2018 growing season similar to that of 2019?**

**Response**: This experiment was performed in a greenhouse with controlled environmental factors, so the plants were not subjected to weather conditions. We have added the word “greenhouse” in the experimental design section to clarify (L 157).

**Line 397–399: In my opinion, the larger the plant is, the larger the plant water consumption will be. The authors should deeply discuss the reason why the water uptake by the plants declined in 2019, because that was unexpected**

**Response**: We agree with the Reviewer that the water uptake declined in 2019 was unexpected. We believe that this could be related to the negative effect of smooth brome on ERC and post oak survival, resource allocation, and total biomass. We discussed this in L 452-461.

**Line 402: Change "regulate" to "affect".**

**Response**: Changed as requested (L 462).

**Line 412: Change "strands" to "stands".**

**Response**: Changed as requested (L 473).

**Line 413: Did you pay attention to the functional traits of smooth brome, such as root length, biomass?**

**Response**: Our study was designed to focus mainly on the impact of ERC although we did present data on smooth brome total biomass in the supplemental material (S1 Fig) that shows no significant differences in total biomass between the different treatments.

**Some relevant references below may be helpful to improve the Discussion:
Barbeta A, and Peñuelas J. 2017. Increasing carbon discrimination rates and depth of water uptake favor the growth of Mediterranean evergreen trees in the ecotone with temperate deciduous forests. Global Change Biology 23:5054–5068.
Bréda N, Huc R, Granier A, and Dreyer E. 2006. Temperate forest trees and stands under severe drought: a review of ecophysiological responses, adaptation processes and long-term consequences. Annals of Forest Science 63:625–644.
Epron D and Dreyer E. 1993. Long-term effects of drought on photosynthesis of adult oak trees [Quercus petraea (Matt.) Liebl. and Quercus robur L.] in a natural stand. New Phytologist 125:381–389.
Liu X, Wang N, Cui R, Song H, Wang F, Sun X, Du N, Wang H, and Wang R. 2021. Quantifying Key Points of Hydraulic Vulnerability Curves From Drought-Rewatering Experiment Using Differential Method. Frontiers in Plant Science 12:627403.
Liu X, Zhang Q, Song M, Wang N, Fan P, Wu P, Cui K, Zheng P, Du N, Wang H, and Wang R. 2021. Physiological Responses of Robinia pseudoacacia and Quercus acutissima Seedlings to Repeated Drought-Rewatering Under Different Planting Methods. Frontiers in Plant Science 12:760510.
Liu Z, Liu Q, Wei Z, Yu X, Jia G, Jiang J. 2021. Partitioning tree water usage into storage and transpiration in a mixed forest. Forest Ecosystems 8:72.
Liu Z, Yu X, Jia G. 2019. Water uptake by coniferous and broad-leaved forest in a rocky mountainous area of northern China. Agricultural and Forest Meteorology 265:381–389.
Montagnoli A, Dumroese RK, Terzaghi M, Onelli E, Scippa GS, and Chiatante D. 2019. Seasonality of fine root dynamics and activity of root and shoot vascular cambium in a Quercus ilex L. forest (Italy). Forest Ecology and Management 431:26–34.
Poca M, Coomans O, Urcelay C, Zeballos SR, Bodé S, Boeckx P. 2019. Isotope fractionation during root water uptake by Acacia caven is enhanced by arbuscular mycorrhizas. Plant and Soil 441:485–497.
Zadworny M, Mucha J, Jagodziński AM, Kościelniak P, Łakomy P, Modrzejewski M, Ufnalski K, Żytkowiak R, Comas LH, and Rodríguez-Calcerrada J. 2021. Seedling regeneration techniques affect root systems and the response of Quercus robur seedlings to water shortages. Forest Ecology and Management 479:118552.**

**Response**: We thank the Reviewer for providing these references. We have cited some of these here including Bréda et al. 2006, Epron and Dreyer, 1993, and Liu et al. 2021.

**Reviewer #2:** **I think this is a well written paper with some interesting results about rooting depth and competitive water extraction. The biggest criticism I have of this paper is the methods used to identify which species was extracting water from specific locations in the soil profile. I think what this design can say is where the water was extracted in the soil profile and not by whom. It is not easy to predict the performance of plants growing in mixtures compared to when they are going alone and root morphological and physiological responses to neighbors may differ from growth in the control, so more direct information is required. Additionally, methods like tracers in soil layers are some useful methods to assess by whom the water is extracted at particular soil layers. I would suggest revising the argument without focusing on the ability to say which species accessed water in which soil layers. Below are additional comments on the manuscripts.**

**Response**: We thank Reviewer#2 for helping us to clarify this manuscript. We have altered the manuscript as requested.

Details:

**Line 6 ERC is colloquial. Rather call it J. virginiana and target species are usually spelled out**

**Response**: We have now altered all reference to ERC and replaced it with *J. virginiana* as requested. We have also altered all reference of post oak and replaced it with *Q. stellata* and smooth brome to *B. inermis*.

**Line 13-14: Is it root overlap & soil water extraction at the same depth? Resources use may not overlap even if roots are?**

**Response**: Plants can use resources from different depths but not beyond their root length. Based on our results in the QUST+BRIN treatment and on our root length data (Fig 2), we can infer that smooth brome mainly relied on water from the top layer of the soil because of their shallow roots and the ERC and post oak, with longer roots, relied on the deeper water. Therefore, there was most probably an overlap in root length and water uptake for ERC and post oak.

**Line 37 requires a citation**.

**Response**: This part was deleted from the Introduction as it does not pertain to the interpretation results of the manuscript.

**Line 38: Change “Reduce” to “reduces”**

**Response**: This part was deleted from the Introduction as it does not pertain to the interpretation results of the manuscript.

**Line 40 citation needed.**

**Response**: Citation added as requested (L 72).

**Line 53: Is this case about tree seedlings?**

**Response**: This is correct. Now added in L 87.

**Line 66: change "are" to "is"**

**Response**: Changed as requested (L 96).

**Line 105: "second" to "the second"**

**Response**: Changed as requested (L 142).

**Line 115: Towards reproducibility of this work: where are the species from? Were the seeds or seedlings purchased or wild collected? When? How were the seedlings propagated for this experiment? About how old were they upon planting?**

**Response**: We purchased our plants from commercial nurseries. *J. virginiana* saplings (two-years old) were obtained from Pinelands Nursery & Supply, New Jersey, *Q. stellata* saplings (one-year old) were obtained from Mossy Oak Nativ Nurseries, Mississippi, and *B. inermis* seeds were obtained from Great Basin Seed company, Utah.

This information is now available in the “Ethics statement” section (L 146-154).

**Line 116 how much time in between planting additional species?**

**Response**: We planted the three species after each other within a two-week span. We first planted the *J. virginiana* saplings, followed by the *Q. stellata*, and finally the *B. inermis* seeds. Now indicated in L 166-169.

**Line 125: Change "plants" to "plant's"**

**Response**: Altered as requested (L 175).

**Line 144 say a little more about FDR**

**Response**: We added more information about FDR (L 185-188).

**Line 146: Where were the access tubes in relation to the plants? Thus, how do you know that the individual species had differential water extraction depths?**

**Response**: The access tubes were installed in the middle of the pots before planting the plants.

We inferred the water extraction depths based on the change in soil moisture content between each treatment and empty pots and based on the species rooting depths (Fig 5).

**Line 182: "Pots with dead ERC were excluded from the analysis." Already said in the methods.**

**Response**: Deleted as requested.

**Line 189 Report statistics here**

**Response**: Statistics included as requested (L 250-254).

**Line 192 the figure 1 legend text is quite long**

**Response**: The figure legend was shortened as requested (L 253-263).

**Line 221 The "ERC" subtitle could be more informative**

**Response**: Altered as requested to read “Water-uptake pattern of *J. virginiana* at different depths” (L 276).

**Line 232 more informative subtitle**

**Response**: Altered as requested to read “Water-uptake pattern of *Q. stellata* at different depths” (L 289).

**Line 234-237: I think what this design can say is where the water was extracted in the soil profile and not by whom. It is not easy to predict the performance of plants growing in mixtures compared to when they are going alone and root morphological and physiological responses to neighbors may differ from growth in the control. Additionally, methods like tracers in soil layers are some useful methods to assess by whom the water is extracted at particular soil layers.**

**Response**: We agree with the Reviewer. We modified our language in the Results and Discussion sections to infer and predict possible shift in soil moisture content in the four treatments and potential water extraction by each species based on our root length data.

**Line 240 "changes" to "changes in"**

**Response**: Altered as requested (L 298).

**Line 246 more informative subheading**

**Response**: Altered as requested to read “Water-uptake pattern of *B. inermis* at different depths” (L 305).

**Line 252-253: Save interpretation for the discussion "This means that the ERC and smooth brome required less water later in the experiment (S1 Table)."**

**Response**: Altered as requested.

**Line 284: The sheer density differences between the grass and the non-target tree has to play a key role here towards growth suppression.**

**Response**: We used the same number of grass seeds and saplings with similar age and height in each pot. We also tried to have similar plant density to that observed in the field.

**Line 353: This is similar to expectations of the limiting similarity hypothesis that was coined after this work. See Fargione & Tilman 2005: Niche differences in phenology and rooting depth promote coexistence with a dominant C4 bunchgrass. Oecologia**

**Response**: Thank you for the suggestion. We added the citation in the Introduction (L 57 and 75).

**Fig 2. legend - make a note that not all species root length was measured in the treatments**

**Response**: Added as requested (L 333).

**Fig. 2 say "different letters" rather than "different symbols"**

**Response**: Altered as requested (L 334).
