# Supplementary material for: Effects of post oak (Quercus stellata) and smooth brome (Bromus inermis) competition on water uptake and root partitioning of eastern redcedar (Juniperus virginiana)
Source: PLoS One. 2023 Feb 1;18(2):e0280100. doi: 10.1371/journal.pone.0280100 (PMC9891534; doi:10.1371/journal.pone.0280100)
Supplement: Attachment — Submitted filename: Response to Reviewers.docx [file pone.0280100.s004.docx]

PONE-D-22-20878R1

Effects of post oak *Quercus stellata* and smooth brome *Bromus inermis* competition on water uptake and root partitioning of eastern redcedar *Juniperus virginiana*

**Responses to the Reviewers:**

We thank the Editor and the Reviewers for their comments and suggestions to improve our

manuscript.

We have made the recommended and suggested changes to our manuscript including simplifying the hypotheses and adding subheadings to the Discussion.

**Reviewer #1: I have read the new version of the manuscript. Authors have rearranged, modified, and shortened the manuscript and responded positively to my comments. Thank the authors for receiving the comments constructively and comprehensively revising the manuscript. Besides, I only have some minor points that need to be clarified and/or corrected.**

**Response:** We thank Reviewer#1 for helping us to clarify this manuscript. We have altered the

manuscript as requested.

Details:

**Line 4: It is better to enclose scientific names in brackets, "Effects of post oak (*Quercus stellata*) and smooth brome (*Bromus inermis*) competition on water uptake and root partitioning of eastern redcedar (*Juniperus virginiana*)".**

**Response**: Thank you for the suggestion. We have altered the title as requested (L 4-6).

**Line 30: Change "two-year old" to "two-year-old".**

**Response**: Altered as requested (L 23).

**Line 166: Change "two-years old" to "two-year-old".**

**Response**: Altered as requested (L 158).

**Line 167: Change "one-year old" to "one-year-old".**

**Response**: Altered as requested (L 159).

**Line 172: Delete the latter "were".**

**Response**: Deleted as requested (L 164).

**Line 191: Change "were" to "was".**

**Response**: Altered as requested (L 183).

**Line 224: Change "different a different amount" to "different amounts".**

**Response**: Altered as requested (L 216).

**Line 234: Change "root-length" to "root length".**

**Response**: Altered as requested (L 226).

**Line 388: Change "suggest" to "suggests".**

**Response**: Altered as requested (L 388).

**Line 466: Change "indicate" to "indicates".**

**Response**: Altered as requested (L 483).

**Reviewer #2: The changes from the previous review have helped the manuscript, but there's still more to clarify. Please see my suggestion below.**

**Response:** We thank Reviewer#2 for helping us clarify this manuscript. We have altered the

manuscript as requested.

Details:

**Line 29-31: treatment summary isn't clear**

**Response**: We have changed and clarified the treatment summary to read “. We had four competition treatments: 1) none, 2) with a native tree (*Quercus stellata*), 3) with an invasive grass (*Bromus inermis*), and 4) with both *Q. stellata* and *B. inermis*” (L24-25).

**Line 97: change "withstand" to "withstands"**

**Response**: Altered as requested (L 90).

**Line 113: change "have" to "has"**

**Response**: Changed as requested (L 107).

**Line 118: change "their" to "its"**

**Response**: Altered as requested (L 112).

**Lines 128 - 143: These hypotheses need to be simplified, made more direct and falsifiable.**

**For example, hypothesis one should read something like: "J. virginiana and B. inermis interactions will lead to water uptake at different depths." The part that reads "because B. inermis should rely on water from the topsoil layers and J. virginiana will invest in belowground development and will extend their roots to get to the deeper water in the soil profile." is something that should be expected given a well-fleshed out introduction, or it should be covered in the discussion if these are the resulting outcomes.**

**Same for all of the hypotheses.**

**Response**: Thank you for the recommendation. We noticed that our hypotheses can be simplified, and we have done so (L 128-135) . The hypotheses are as follows:

1) *J. virginiana* and *B. inermis* interactions will lead to water uptake at different depths.

2) There will be niche separation between *J. virginiana* and *Q. stellata* roots [31].

3) Soil moisture will be consistently low throughout the soil profile when all three species (*J. virginiana*, *Q. stellata*, *B. inermis*) co-occur, leading to lower water potential for *J. virginiana*.

4) We expect that the water requirements for *J. virginiana*, *Q. stellata*, and *B. inermis* will increase with time as the plants grow, leading to more intense water stress in the second year of the experiment.

**line 185: change "measure" to "measures"**

**Response**: Changed as requested (L 177).

**Line 224: remove "different"**

**Response**: Deleted as requested (L 216)

**Line 238: towards reproducibility: clarify which tests were run in R and which in SPSS**

**Response**: Thank you for the recommendation. We have clarified which tests were run in R and which in SPSS to read “The LOESS regression and figures were all done in R version 3.6.0 (R Development Core Team, 2019). All other statistical analyses were done in SPSS 26.0 statistical package (IBM SPSS, 2019)” (L 229-231).

**Line 251-252: Give this sentence a little more care and indicate the p value for each.**

**Response**: Thank you for the suggestion. All p values were significant (p < 0.001). The information in that sentence was presented in S1 Table. Therefore for clarity we eliminated the confusing sentence and now, refer to that table and include “All regression lines were significant (p < 0.001)” (L 224 ) and in S1 Table.

**Table 1: tables and figures should be interpretable without reference to the text. So, the treatment acronyms require explanation. Clarify that QUST+BRIN contains 3 species**

**Response**: Thank you for the comment. We have included an explanation of the treatment acronyms for all tables and graphs.
“JUVI *=* *J. virginiana* only, QUST *=* *J. virginiana* growing with *Q. stellata*, BRIN *= J. virginiana* growing with *B. inermis*, and QUST+BRIN *=* *J. virginiana* growing with both *Q. stellata* and *B. inermis*.”

**Line 277: save "As predicted" for the discussion**

**Response**: Deleted as requested.

**line 278-280: re: "likely" - they either did or they didn't; and "due to their extensive rooting system" this interpretation is better left for the discussion since this wasn't tested directly.**

**Response**: We removed this interpretation from the results and included it in the Discussion (L 399-400).

**Line 292 -294: Save for the discussion "We can likely attribute this decline in moisture content at the shallow depths to water uptake by Q. stellata because there was no significant change in water uptake at 30 cm and 40 cm compared to the JUVI treatment"**

**Response**: We deleted the interpretation from the Results.

**Line 307-308: save interpretation for the discussion "We can attribute this decline to the increase in water demand, mainly to the presence of B. inermis."**

**Response**: We deleted the interpretation from the Results and moved it to the Discussion.

**Line 309: "in contrast" and "dissension" are redundant**

**Response**: We deleted “in dissention” (L 299).

**Line 329: save for the discussion "potentially due to the overlap in J. virginiana and Q. stellata rooting depths."**

**Response**: We deleted the interpretation from the Results section.

**Line 382: One of the previous reviewers suggested adding subheadings to the Discussion and I think this is needed for clarity.**

**Response:** Thank you for the suggestion. We have added subheadings to the Discussion (L 394, 407, 458, and 477).

**Line 384: rephrase "co-exist for water", e.g., "compete for water"**

**Response**: Altered as requested (L 384).

**Line 407-411: this is unknown**

**Response**: We acknowledge that our study design does not allow us to draw this conclusion from our data and we have added text to the discussion to clarify this fact (L 422-426). This is also relevant for the subsequent comment about L 408.

**Line 408: It sounds like you have the data to graph how much water was extracted from the different soil depths to better make this conclusion - like a density plot or a violin plot.**

**Response**: The FDR method we used to characterize soil moisture depletion does not allow us to quantify the total amount of water taken up at each depth, rather it gives us a relative measure of soil water depletion across the different depths. Given the interesting patterns described above, water consumption of *J. virginiana* competition should be addressed in future studies to better understand the full impact of water competition on establishment.

**line 463: clarify "indirectly as fuel for fires" to you mean the grass-fueled fires destroy saplings?**

**Response**: We have clarified this sentence to read “Grasses can also have an indirect effect as grass-fueled fires can destroy saplings” (L 479-480).
